# Supplementary material for: Retinal biomarkers of Cerebral Small Vessel Disease: A systematic review
Source: PLoS One. 2022 Apr 14;17(4):e0266974. doi: 10.1371/journal.pone.0266974 (PMC9009626; doi:10.1371/journal.pone.0266974)
Supplement: S1 File — Terms, syntax, and databases used to perform systematic review of scientific literature. (DOCX) [file pone.0266974.s002.docx]

**Retinal Imaging Biomarkers of Cerebral Small Vessel Disease:**

**a Systematic Review**

EZ Biffi et al.

**SEARCH STRATEGY**

***Search Terms***

| Terms Group 1 (CSVD): | Terms Group 2 (Retinal Imaging): |
| --- | --- |
| 1. **MeSH terms:**  - "Cerebral Small Vessel Diseases"[Mesh] - "CADASIL"[Mesh] - "Cerebral Amyloid Angiopathy"[Mesh] - “MELAS Syndrome”[Mesh]  1. **Title/Abstract Terms**   **(Free text terms):**   - ”Cerebral microvasculature”[tiab] - ”Cerebral amyloid angiopathy”[tiab] - ”Cerebral autosomal dominant arteriopathy with subcortical infarcts and leukoencephalopathy”[tiab] - ”CADASIL”[tiab] - ”Mitochondrial encephalopathy lactic acidosis and stroke like episodes”[tiab] - ”MELAS”[tiab] - ”Cerebral small vessel disease”[tiab] - “CSVD”[tiab] - ”Hypertensive arteriolosclerosis”[tiab] - ”Hypertension related angiopathy””[tiab] | 1. **MeSH Terms:**  - "Tomography, Optical Coherence"[Mesh] - "Electroretinography"[Mesh] - "Optical Imaging"[Mesh] - “Visual field tests”[Mesh  1. **Title/Abstract Terms**   **(Free text terms):**   - “Fundus photography”[tiab] - “Fundus autofluorescence”[tiab] - “Optical coherence tomography angiography”[tiab] - “OCT Angiography”[tiab] - “Optical Coherence Tomography”[tiab] - “OCT”[tiab] - “Electroretinography”[tiab] - “Optical Imaging”[tiab] - “Visual field”[tiab] - “Automated perimetry”[tiab] |

***Records Inclusion Criteria:***

**Inclusion criteria for the abstract/title screening through the database search**:

1. **Topic 1: Cerebral SVD**, 5 types:
   1. Sporadic: Hypertensive arteriolosclerosis or hypertension-related angiopathy (HTNA) and cerebral amyloid angiopathy (CAA)
   2. Genetic: Fabry disease, Cerebral Autosomal Dominant Arteriopathy with Subcortical Infarcts and Leukoencephalopathy (CADASIL), and Mitochondrial Encephalopathy, Lactic acidosis, and Stroke-like episodes (MELAS) syndrome
2. **Topic 2: Retinal imaging**, including:
3. Fundus Photography (standard camera, Wide-field)
4. OCT (Spectral Domain SD-OCT, Swept Source SS-OCT)
5. OCT-Angiography (OCTA)
6. Fluorescein Angiography
7. Visual Field
8. ERG
9. Visual Evoked Potential
10. **Subjects:** only human studies
11. **Language:** only English
12. **Study design:** only peer-reviewed, case-based (more than 1 case) and case-control studies. Exclusion: conference abstracts and summaries, single case report, review articles
13. **Quantification analysis:** was performed

***Final Search Strategies***

PubMed Search Strategy

((((Cerebral Small Vessel Diseases[MeSH Terms] OR CADASIL[MeSH Terms] OR Cerebral Amyloid Angiopathy[MeSH] OR MELAS Syndrome[MeSH Terms**]**) OR (“cerebral small vessel disease*"[Title/Abstract] OR “retinal vessels”[Title/Abstract] OR CSVD[Title/Abstract] OR "cerebral microvasculature"[Title/Abstract] OR CADASIL[Title/Abstract] OR “cerebral amyloid angiopathy”[Title/Abstract] OR MELAS[Title/Abstract] OR "cerebral autosomal dominant arteriopathy with subcortical infarcts and leukoencephalopathy"[Title/Abstract] OR “mitochondrial encephalopathy lactic acidosis and stroke like episodes”[Title/Abstract] OR “hypertensive arteriolosclerosis”[Title/Abstract] OR “hypertension related angiopathy”[Title/Abstract] OR “Fabry Disease”[Title/Abstract])) AND (("Tomography, Optical Coherence"[MeSH Terms] OR "Electroretinography"[MeSH Terms] OR "Optical Imaging"[MeSH Terms] OR “Visual Field Tests”[MeSH Terms]) OR ("optical coherence tomography"[Title/Abstract] OR OCT[Title/Abstract] OR "fundus autofluores*"[Title/Abstract] OR "fundus photography"[Title/Abstract] OR “optical coherence tomography angiography”[Title/Abstract] OR “OCT angiography”[Title/Abstract] OR OCTA[Title/Abstract] OR electroretinography[Title/Abstract] OR "visual field"[Title/Abstract] OR "optical imaging"[Title/Abstract] OR "automated perimetry*"[Title/Abstract]))) NOT (("Macular Degeneration"[MeSH Terms] OR “Central Serous Chorioretinopathy"[MeSH Terms] OR Glaucoma[MeSH Terms] OR "Retinal Vein Occlusion"[MeSH Terms] OR "Retinal Detachment"[MeSH Terms] OR Uveitis[MeSH Terms] OR "Retinitis Pigmentosa"[MeSH Terms]) OR ("macular degeneration"[Title/Abstract] OR "central serous chorioretinopath*"[Title/Abstract] OR glaucoma [Title/Abstract] OR "retinal vein occlusion"[Title/Abstract] OR "retinal detachment"[Title/Abstract] OR uveitis[Title/Abstract] OR "retinitis pigmentosa"[Title/Abstract]))) AND ((clinicalstudy[Filter] OR clinicaltrial[Filter] OR clinicaltrialphaseI[Filter] OR clinicaltrialphaseII[Filter] OR clinicaltrialphaseIII[Filter] OR clinicaltrialphaseIV[Filter] OR comparativestudy[Filter] OR controlledclinicaltrial[Filter] OR englishabstract[Filter] OR evaluationstudy[Filter] OR journalarticle[Filter] OR multicenterstudy[Filter] OR observationalstudy[Filter] OR pragmaticclinicaltrial[Filter] OR review[Filter] OR meta-analysis[Filter] OR randomizedcontrolledtrial[Filter] OR validationstudy[Filter] OR systematicreview[Filter]) AND (humans[Filter]) AND (english[Filter]))

Ovid MEDLINE Search Strategy

((Cerebral Small Vessel Diseases/ or CADASIL/ or Cerebral Amyloid Angiopathy/ or MELAS Syndrome/ or (cerebral-small-vessel-disease* or retinal-vessels or CSVD or cerebral-microvasculature or CADASIL or cerebral-amyloid-angiopathy or MELAS or cerebral-autosomal-dominant-arteriopathy-with-subcortical-infarcts-and-leukoencephalopathy or mitochondrial-encephalopathy-lactic-acidosis-and-stroke-like-episodes or hypertensive-arteriolosclerosis or hypertension-related-angiopathy or Fabry-Disease).ab,ti.) and (Tomography, Optical Coherence/ or Electroretinography/ or Optical Imaging/ or Visual Field Tests/ or (optical-coherence-tomography or OCT or fundus-autofluores* or fundus-photography or optical-coherence-tomography-angiography or OCT-angiography or OCTA or eletroretinography or visual-field or optical-imaging or automated-perimetr*).ab,ti.)) not (Macular Degeneration/ or Central Serous Chorioretinopathy/ or Glaucoma/ or Retinal Vein Occlusion/ or Retinal Detachment/ or Uveitis/ or Retinitis Pigmentosa/ or (macular-degeneration or central-serous-chorioretinopath* or glaucoma or retinal-vein-occlusion or retinal-detachment or uveitis or retinitis-pigmentosa).ab,ti.)

Scopus Search Strategy

( TITLE-ABS-KEY ( ( "cerebral small vessel disease*" OR "retinal vessels" OR "CSVD" OR "cerebral microvasculature" OR "CADASIL" OR "cerebral amyloid angiopathy" OR "MELAS" OR “cerebral autosomal dominant arteriopathy with subcortical infarcts and leukoencephalopathy” OR “mitochondrial encephalopathy lactic acidosis and stroke like episodes” OR "hypertensive arteriolosclerosis" OR "hypertension related angiopathy" OR "Fabry Disease" ) AND ( "optical coherence tomography" OR oct OR "fundus autofluores*" OR "fundus photography" OR "optical coherence tomography angiography" OR "OCT angiography" OR octa OR "electroretinography" OR "visual field" OR "optical imaging" OR "automated perimetry*" ) ) AND NOT ( "macular degeneration" OR "central serous chorioretinopath*" OR "glaucoma" OR "retinal vein occlusion" OR "retinal detachment" OR "uveitis" OR "retinitis pigmentosa" ) ) AND ( LIMIT-TO ( DOCTYPE , "ar" ) OR LIMIT-TO ( DOCTYPE , "re" ) ) AND ( LIMIT-TO ( SUBJAREA , "MEDI" ) OR LIMIT-TO ( SUBJAREA , "HEAL" ) ) AND ( LIMIT-TO ( LANGUAGE , "English" ) )

Cochrane Library Search Strategy

Line 1: Go to Search manager tab. In line #1, click MeSH. In the “Enter MeSH term” box that appears, type *Cerebral Small Vessel Diseases*, then Look up. Make sure “expode all trees” is selected, then click Add/edit search line.

Lines 2-4: Repeat this for the MeSH terms *CADASIL* (make sure to use all caps for CADASIL), *Cerebral Amyloid Angiopathy*, and *MELAS Syndrome* on the following search lines, respectively.

Line 5: Click the S button. In the Title/Abstract/Keyword search box, Cut and paste the following into #5, put quotes around it, and click Add/Edit search line:

*cerebral small vessel disease*

Lines 6-16: Repeat this for each of the following search terms, respectively (note that *CSVD*, *CADASIL*, and *MELAS* do not need quotes):

*retinal vessels, CSVD, cerebral microvasculature, CADASIL, cerebral amyloid angiopathy, MELAS, cerebral autosomal dominant arteriopathy with subcortical infarcts and leukoencephalopathy, mitochondrial encephalopathy lactic acidosis and stroke like episodes, hypertensive arteriolosclerosis, hypertension related angiopathy, Fabry Disease*

Lines 17-20: Repeat the process for Line 1 for the following MeSH terms, respectively:

*Tomography, Optical Coherence, Electroretinography, Optical Imaging, Visual Field Tests*

Lines 21-31: Repeat the process for Line 5 for the following terms, respectively (do not include quotes for *OCT*, *fundus NEAR autofluores**, *OCTA*, *electroretinography*, or *automated NEAR perimetry**):

*optical coherence tomography, OCT, fundus NEAR autofluores*, fundus photography, optical coherence tomography angiography, OCT angiography, OCTA, electroretinography, visual field, optical imaging, automated NEAR perimetr**

Lines 32-38: Repeat the process for Line 1 for the following MeSH terms, respectively:

*Macular Degeneration, Central Serous Chorioretinopathy, Glaucoma, Retinal Vein Occlusion, Retinal Detachment, Uveitis, Retinitis Pigmentosa*

Lines 39-45: Repeat the process for Line 5 for the following terms, respectively (do not include quotes for *central NEAR serous NEAR chorioretinopath**, *glaucoma*, or *uveitis*):

*macular degeneration, central NEAR serous NEAR chorioretinopath*, glaucoma, retinal vein occlusion, retinal detachment, uveitis, retinitis pigmentosa*

Line 46: Cut and paste the following:

{OR #1-#16} AND {OR #17-#31}

Line 47: Cut and paste the following:

#46 NOT {OR #32-#45}

Web of Science Search Strategy

(TS=(“cerebral small vessel disease*” OR “retinal vessels” OR CSVD OR “cerebral microvasculature” OR CADASIL OR “cerebral amyloid angiopathy” OR MELAS OR “cerebral autosomal dominant arteriopathy with subcortical infarcts and leukoencephalopathy” OR “mitochondrial encephalopathy lactic acidosis and stroke like episodes” OR “hypertensive arteriolosclerosis” OR “hypertension related angiopathy” OR “Fabry Disease”) AND TS=(“optical coherence tomography” OR OCT OR “fundus autofluores*” OR “fundus photography” OR “optical coherence tomography angiography” OR “OCT angiography” OR OCTA OR electroretinography OR “visual field” OR “optical imaging” OR “automated perimetr*”)) NOT TS=(“macular degeneration” OR “central serous chorioretinopath*” OR glaucoma OR “retinal vein occlusion” OR “retinal detachment” OR uveitis OR “retinitis pigmentosa”)

Along the left sidebar, filter by Language > English and click Refine
